# Personality and change in perceived control across the first four months of the coronavirus pandemic

**DOI:** 10.1093/geroni/igab046.2691

**Published:** 2021-12-17

**Authors:** Amanda Sesker, Ji Hyun Lee, Martina Luchetti, Damaris Aschwanden, Yannick Stephan, Antonio Terracciano, Angelina Sutin

**Affiliations:** 1 Florida State University College of Medicine, Tallahassee, Florida, United States; 2 University of Michigan, Ann Arbor, Michigan, United States; 3 Florida State University, Tallahassee, Florida, United States; 4 University of Montpellier, Montpellier, Languedoc-Roussillon, France; 5 FLORIDA STATE UNIVERSITY, Florida State University, Florida, United States

## Abstract

Objective. This study examined change in perceived control (PC) across the first four months of the global coronavirus pandemic and whether change varied significantly by age and personality traits during the first four months of the pandemic. Methods. Personality was assessed prior to the pandemic in a large national sample of 2,455 American adults (18-100 years) from a preregistered online survey (https://osf.io/q8cpd), first conducted between January 31, 2020 and February 10, 2020. Three additional follow-up waves were conducted: mid-March 2020 (following the World Health Organization declaration of the coronavirus a pandemic), late April 2020 (toward the end of the White House’s 30 Days to Slow the Spread guidance), and late July 2020 (when patient deaths in the U.S. reached 132,918). PC was assessed in Waves 2-4. Results. There were age differences in the trajectory of PC such that PC increase for middle-aged and older adults, whereas younger adults had lower PC and did not increase over the follow-ups. All personality traits but Openness were associated with PC at the first assessment. Conscientiousness, Extraversion, and Agreeableness positively predicted PC change over time. The association between Conscientiousness and Extraversion and increased PC over time was stronger at older ages. Conclusion. Pre-pandemic personality predicted PC and PC change during the first four months of the pandemic, with middle-aged and older adults showing better adaption than younger adults. This study provides new evidence for PC change and associations between personality and PC during the COVID-19 pandemic.

